# YOLO11m-Based Artificial Intelligence Versus Orthopaedic Physician Panels for Arthroscopy-Referenced Three-Category MRI Classification of the Posterosuperior Rotator Cuff: A Multi-Reader Diagnostic Accuracy Study

**DOI:** 10.3390/diagnostics16213535

**Published:** 2026-10-28

**Authors:** Halil Büyükdoğan, Tahir Burak Sarıtaş, Avni Tan, Abdülkadir Yalçınkaya, Erhan Abay, Muhammed Halit Yücel, Volkan Arıcı, Necati Doğan, Adem Şahin, Cemil Ertürk

**Affiliations:** 1 Department of Orthopaedics and Traumatology, Gaziosmanpaşa Hospital, Faculty of Medicine, Istinye University, Istanbul 34245, Türkiye; 2 Department of Orthopaedics and Traumatology, Kanuni Sultan Süleyman Training and Research Hospital, University of Health Sciences, Istanbul 34303, Türkiye; tahirburak.saritas@sbu.edu.tr (T.B.S.); dr.avnitan@gmail.com (A.T.);; 3 Department of Orthopaedics and Traumatology, Faculty of Medicine, Istanbul Yeni Yüzyıl University, Istanbul 34010, Türkiye

**Keywords:** artificial intelligence, deep learning, magnetic resonance imaging, rotator cuff tears, arthroscopy, diagnostic accuracy, observer variability

## Abstract

**Background/Objectives:** Artificial intelligence (AI) may support rotator cuff MRI interpretation; however, its performance relative to clinicians at different experience levels remains uncertain. We compared a locked YOLO11m-based model with prespecified orthopaedic physician panels using arthroscopy as the reference standard. **Methods:** This retrospective single-centre study included a separate 300-patient model-development dataset and an arthroscopy-referenced reader cohort of 258 patients (60 intact cuffs, 86 partial-thickness tears, and 112 full-thickness tears). Twelve blinded physicians were assigned to four three-reader experience panels, and panel diagnoses were defined by a two-of-three majority. Paired accuracy comparisons used exact McNemar tests with Holm adjustment. **Results:** AI achieved 0.841 overall accuracy (95% CI, 0.792–0.881). The senior-specialist panel had numerically higher accuracy (0.911), but the difference from AI was not statistically significant after adjustment (Holm *p* = 0.060); the junior-specialist panel also did not differ from AI (0.822; Holm *p* = 0.649). AI showed higher paired accuracy than the senior-resident (0.729; Holm *p* = 0.005) and junior-resident panels (0.671; Holm *p* < 0.001). Partial-thickness tears had the lowest AI sensitivity (0.709). **Conclusions:** In this restricted-cine, three-category benchmark, AI outperformed both resident panels, whereas neither specialist panel comparison reached the adjusted significance threshold. These findings do not establish equivalence to specialists or benefit from AI-assisted reading.

## 1. Introduction

Rotator cuff tears are a common cause of shoulder pain and functional impairment, encompassing a spectrum of partial-thickness defects to full-thickness tendon disruption. Accurate characterisation of rotator cuff integrity is clinically important because tear severity may influence treatment planning and surgical decision-making. Although magnetic resonance imaging (MRI) is central to noninvasive rotator cuff assessment, differentiating between intact tendons, partial-thickness tears, and full-thickness tears can be challenging, particularly when structural abnormalities are subtle or incomplete [1,2].

Artificial intelligence (AI) has gained increasing attention in shoulder and elbow surgery, particularly for imaging-based diagnosis, segmentation, and outcome prediction, with rotator cuff MRI representing a major area of application [3,4,5]. Most published models have focused on the supraspinatus, using binary, multiclass, or multitask frameworks; fewer have assessed other rotator cuff tendons or compared AI with human readers [6,7,8,9]. A recent systematic review of MRI-based AI models also demonstrated substantial heterogeneity in imaging protocols, model architectures, reference standards, and human-comparator designs [10]. Deep learning has also been applied to automated three-dimensional segmentation and quantification of posterosuperior full-thickness tears [11]. Arthroscopy-referenced studies have evaluated tear status and size, partial- versus full-thickness staging, and detailed grading of partial-thickness tears [12,13,14]. More recent patient-level and multicentre studies have extended three-category classification and reader comparisons, although surgical validation was not consistently available and some classifications combined partial-thickness tears with tendinopathy [15,16,17]. Despite promising results, these studies remain heterogeneous in tendon coverage, class definitions, reference standards, and reliability assessment. Prior studies have not consistently combined an arthroscopic reference standard, patient-level three-category classification, comparisons across predefined orthopaedic experience levels, and descriptive analysis of first-pass physician-panel agreement.

With arthroscopic findings used to establish the reference classification of posterosuperior rotator cuff status as intact, partial-thickness tear, or full-thickness tear, the primary aim of this study was to evaluate the overall and class-specific diagnostic performance of a locked YOLO11m model in interpreting preoperative coronal T2-weighted fat-suppressed MRI sequences and to compare this performance with four prespecified three-reader orthopaedic physician panels representing different levels of clinical experience. The secondary aim was to describe first-pass agreement within physician panels. We hypothesised that the AI performance relative to the physician panels would vary by experience level and tear category, with the largest relative advantage for AI expected in the resident panels.

## 2. Materials and Methods

### 2.1. Study Design and Patient Selection

This retrospective, single-centre study comprised two non-overlapping cohorts: a separate 300-patient MRI-based model-development cohort and an arthroscopy-referenced diagnostic reader cohort of 258 patients. A stratified subset of 39 cases from the reader cohort (13 from each arthroscopic reference category) was reassessed for reader reliability. The reader-study cohort consisted of consecutive eligible shoulder arthroscopy cases treated between January 2019 and December 2025. Ethical approval was obtained from the Kanuni Sultan Süleyman Training and Research Hospital Clinical Research Ethics Committee (Approval No. KAEK/2026.04.168; 22 April 2026). The study adhered to the Declaration of Helsinki; written informed consent was waived because the research used retrospective anonymised imaging and operative data.

Patients were eligible for the 258-case diagnostic accuracy study if they had undergone shoulder arthroscopy, had a preoperative shoulder MRI containing an assessable coronal T2-weighted fat-suppressed sequence, and had complete MRI and operative documentation permitting a three-category posterosuperior rotator cuff classification. Consecutive eligible patients were included without category-based sampling, and each patient contributed one operated shoulder and one corresponding preoperative MRI examination. The exact MRI-to-arthroscopy interval was not retained as a separate analysable variable in the study dataset and, therefore, could not be reconstructed reliably for this revision.

**Figure 1:**
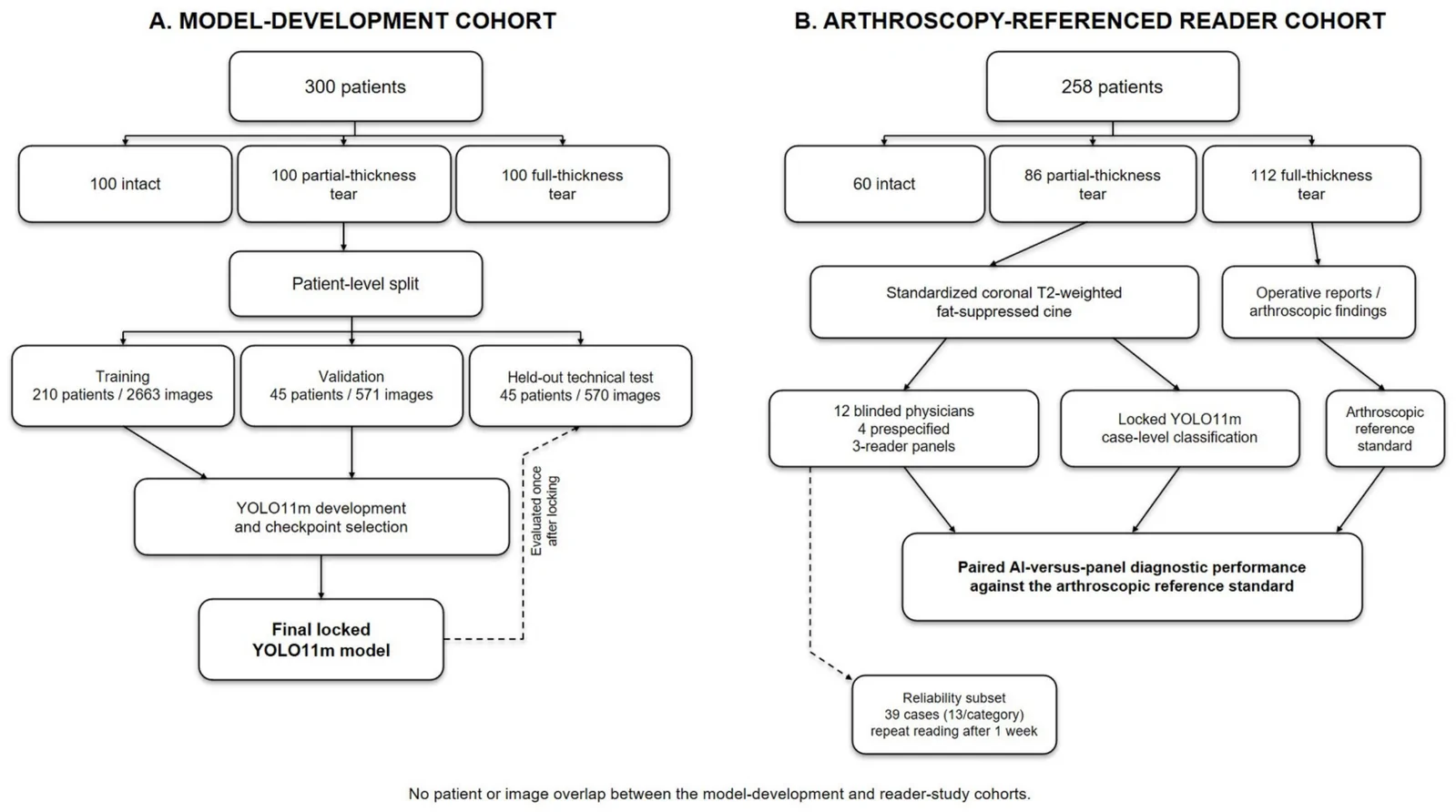
The study design and cohort relationships. The separate MRI-based model-development cohort comprised 300 patients and was divided at the patient level into training, validation, and held-out technical test subsets. Model development and checkpoint selection used the training and validation subsets, after which the final locked YOLO11m model was evaluated once on the held-out technical test subset. The arthroscopy-referenced reader cohort comprised 258 patients and was evaluated independently by the locked YOLO11m model and 12 blinded physicians organised into four prespecified three-reader panels, using the arthroscopic classification as the reference standard. A stratified 39-case subset of the reader cohort (13 cases per arthroscopic reference category) underwent repeat reading after approximately one week for reliability assessment. There was no patient or image overlap between the model-development and reader-study cohorts.

The exclusion criteria were previous surgery on the same shoulder, inadequate image quality, incomplete MRI, absence of the required MRI sequence, missing or insufficient operative documentation, fracture, infection, tumour, and advanced arthropathy preventing reliable rotator cuff assessment. The final reader-study cohort comprised 258 eligible patients: 60 with an intact posterosuperior cuff, 86 with a partial-thickness tear, and 112 with a full-thickness tear. The available analytic dataset did not preserve a reliable denominator of all initially screened arthroscopy cases or detailed surgical-indication variables; therefore, these were not reconstructed post hoc. The study design and cohort relationships are summarised in Figure 1.

Clinical indications for arthroscopy were heterogeneous, including unsuccessful nonoperative treatment, acute or extensive rotator cuff pathology, and clinically important functional impairment. An intact posterosuperior cuff at arthroscopy did not exclude another operative indication, such as refractory adhesive capsulitis. These examples provide clinical context rather than patient-level frequencies, as detailed indications were not retained in the analytic dataset.

### 2.2. Arthroscopic Reference Standard

The arthroscopic reference standard was abstracted from the operative reports. The surgeon who performed each procedure confirmed the operative classification, and the articular and bursal surfaces of the posterosuperior cuff were assessed arthroscopically. MRI findings did not substitute for the operative description when assigning the reference category. The operating surgeons had access to the preoperative MRI during routine clinical care and therefore were not blinded to its findings. This may have introduced expectation or review bias in the assessment and documentation of the operative reference standard.

The posterosuperior rotator cuff was defined as the supraspinatus, infraspinatus, and teres minor tendons. A full-thickness tear was assigned when any of these tendons had a defect extending through the full tendon thickness; a partial-thickness tear was assigned in the absence of a full-thickness lesion when an incomplete-thickness defect affected at least one tendon; and an intact cuff was assigned when no tear was documented in the three posterosuperior tendons. The study endpoint was tear integrity rather than tendinopathy or detailed tear grading; tendon-specific sidedness, depth, retraction, and size were not analysed as separate diagnostic endpoints. Concomitant isolated subscapularis pathology did not by itself determine the posterosuperior category. A separate adjudication procedure for equivocal arthroscopic findings and a dedicated classification rule for isolated intratendinous abnormalities could not be verified from the retained study records; these procedures were not reconstructed retrospectively.

Reference categories were finalised from the arthroscopy records before the AI inference and blinded reader assessment. The 12 readers and AI model had no access to operative records or arthroscopic reference labels during image interpretation. None of the 12 diagnostic readers participated in the development of the annotations.

### 2.3. MRI Acquisition and Cine Loop Preparation

All preoperative shoulder MRI examinations were performed using a 1.5 T MAGNETOM Aera system (Siemens Healthineers, Erlangen, Germany) with a dedicated shoulder coil. Imaging was performed using the institution’s standardised coronal T2-weighted fat-suppressed shoulder protocol, with 17–21 consecutive source slices per sequence. Numerical acquisition parameters, including repetition and echo times, field of view, acquisition matrix, slice thickness, and inter-slice gap, could not be verified from the retained analytic extract and are therefore not reported as observed study measurements.

The complete coronal T2-weighted fat-suppressed series was retrieved from the institutional picture archiving and communication system and anonymised prior to analysis. The ordered source images were converted into a standardised cine for both physician assessment and AI application. According to the authors’ retained technical description, cine preparation used Python 3.10 and OpenCV 4.8 with images standardised to 640 × 640 pixels (window centre, 400; window width, 800). Each 10 s playback was encoded at 30 frames per second (300 encoded frames) and displayed twice, producing 600 encoded frames over 20 s from only 17–21 original MRI source slices. Several consecutive encoded frames could therefore represent the same anatomical source slice; a video-frame index was not equivalent to an anatomical slice identifier. DICOM identifiers and position metadata provide the intended basis for distinguishing and ordering the anatomical source slices, as illustrated by the example in Supplementary Figure S1 (CASE-FT-001). The complete original export and inference code was not available for independent verification of this mapping across all clinical cases.

For observer assessment, the sequence was displayed twice consecutively for a total viewing time of 20 s and could not be paused, magnified, slowed, or manually navigated. After the second presentation, the observers had up to 10 s to record one patient-level diagnosis. The physicians and AI were evaluated from the same coronal T2-weighted fat-suppressed source sequence; neither had access to additional MRI sequences, clinical information, radiology reports, arthroscopic findings, or operative records during the study evaluation.

### 2.4. Model Development and Technical Testing

The AI model was developed using the YOLO11m segmentation architecture initialised from a Common Objects in Context pretrained segmentation checkpoint. The separate development dataset comprised 3804 coronal T2-weighted fat-suppressed shoulder MRI images from 300 patients with nonsurgical management, including 100 intact cuffs, 100 partial-thickness tears, and 100 full-thickness tears. The development cohort had no patient or image overlap with the arthroscopy-referenced reader-study cohort.

Each source sequence contained 17–21 slices. Peripheral slices without an evaluable relationship between the posterosuperior rotator cuff and tendon footprint were excluded, leaving approximately 12–13 diagnostically relevant images per patient for analysis. The model was trained to assess the overall posterosuperior cuff–footprint relationship rather than individual tendons.

Data were split at the patient level to prevent information leakage, with stratified allocation of 70% for training, 15% for validation, and 15% for held-out technical testing. The training subset comprised 210 patients and 2663 images, the validation subset comprised 45 patients and 571 images, and the technical test subset comprised 45 patients and 570 images, with equal representation of the three diagnostic categories. No reader-study case contributed to model training, validation, technical testing, threshold selection, or model development.

Before training, automatic orientation correction was applied, and images were resized to 640 × 640 pixels. Data augmentation was restricted to the training subset and included random rotation within 15 degrees, horizontal and vertical shear up to 10 degrees, and image blurring up to 0.8 pixels. No augmentation was applied to the validation or technical test subsets.

Class-specific instance-segmentation annotations were created in the Computer Vision Annotation Tool (CVAT) by three orthopaedic specialists with more than five years of clinical experience. Diagnostic labels and segmentation masks were established through joint consensus review according to the tendon–footprint relationship. The annotators had access to complete multiplanar MRI examinations when assigning MRI-consensus categories, while only selected coronal T2-weighted fat-suppressed images were retained as model inputs. Joint annotation did not involve three independent mask sets or a separate formal adjudication process; therefore, independent annotation-disagreement rates cannot be reported. Because the development cohort was nonsurgically managed, these labels were MRI-consensus labels and were not arthroscopically verified. Individual post-specialist years and the shoulder-specific MRI-reading experience of the three annotators were not recorded separately in the development dataset and were therefore not reconstructed retrospectively. None of the 12 reader-study observers participated in the annotation.

**Figure 2:**
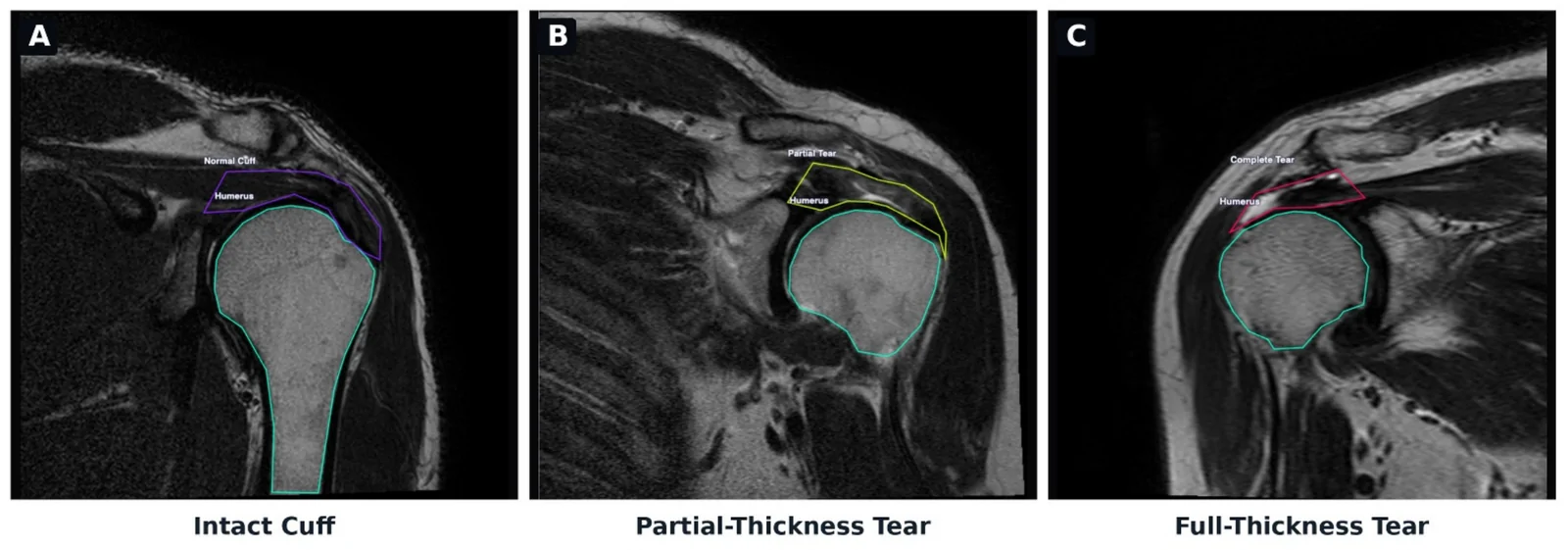
Representative CVAT annotations supplied by the authors from the MRI-consensus model-development cohort: (**A**) Normal Cuff, (**B**) Partial Tear, and (**C**) Complete Tear. Each example shows the class-labelled composite cuff–footprint contour and the auxiliary Humerus contour. These are development annotations, not AI-generated predictions. Humerus contributed to the pooled four-class technical segmentation metrics but was excluded from patient-level tear classification.

The implemented segmentation output included three diagnostic classes (Normal Cuff, Partial Tear, and Complete Tear) and an auxiliary Humerus localisation class (Figure 2). The three diagnostic classes shared a composite cuff–footprint mask encompassing the visible posterosuperior tendon and its humeral attachment, including the visible tear region when present; individual tendons, the footprint, and the defect were not segmented as separate classes. Normal Cuff indicated no visible tear, Partial Tear indicated a defect involving only part of tendon thickness, and Complete Tear indicated full-thickness disruption extending from the articular to the bursal surface. The auxiliary Humerus mask outlined the visible humerus as an anatomical localisation target. All four classes contributed to pooled technical segmentation-mask metrics; the Humerus class was excluded from three-category patient-level tear classification. In tear-positive examinations, slices without the category-defining tear appearance were not retained as Normal Cuff training images; therefore, the model-development task remained a curated segmentation task rather than an unrestricted whole-examination classification.

Training was performed for a maximum of 100 epochs using a batch size of 16, AdamW optimiser, initial learning rate of 0.001, and early stopping with a patience of 20 epochs. The model was implemented using Ultralytics version 8.3.0 and PyTorch version 2.11.0 in Python 3.10, and trained on an NVIDIA GeForce RTX 4090 graphics processing unit with 24 GB of memory. A fixed random seed of 42 was employed.

The final checkpoint was selected exclusively according to the highest validation mask mean average precision at an intersection-over-union threshold of 0.50. Technical performance was then assessed once on the held-out technical test subset using mask mean average precision at 0.50, precision, recall, and F1 score. The technical test subset was not used for model selection, hyperparameter optimisation, early stopping, or recalibration. Because this subset used MRI-consensus development labels rather than arthroscopy, it was reported as a technical segmentation test and not as a second clinical diagnostic-validation cohort. A separately verified patient-level three-category classification summary for this 45-patient technical test subset was not retained and is therefore not reported.

### 2.5. Artificial Intelligence Model and Case-Level Classification

**Figure 3:**
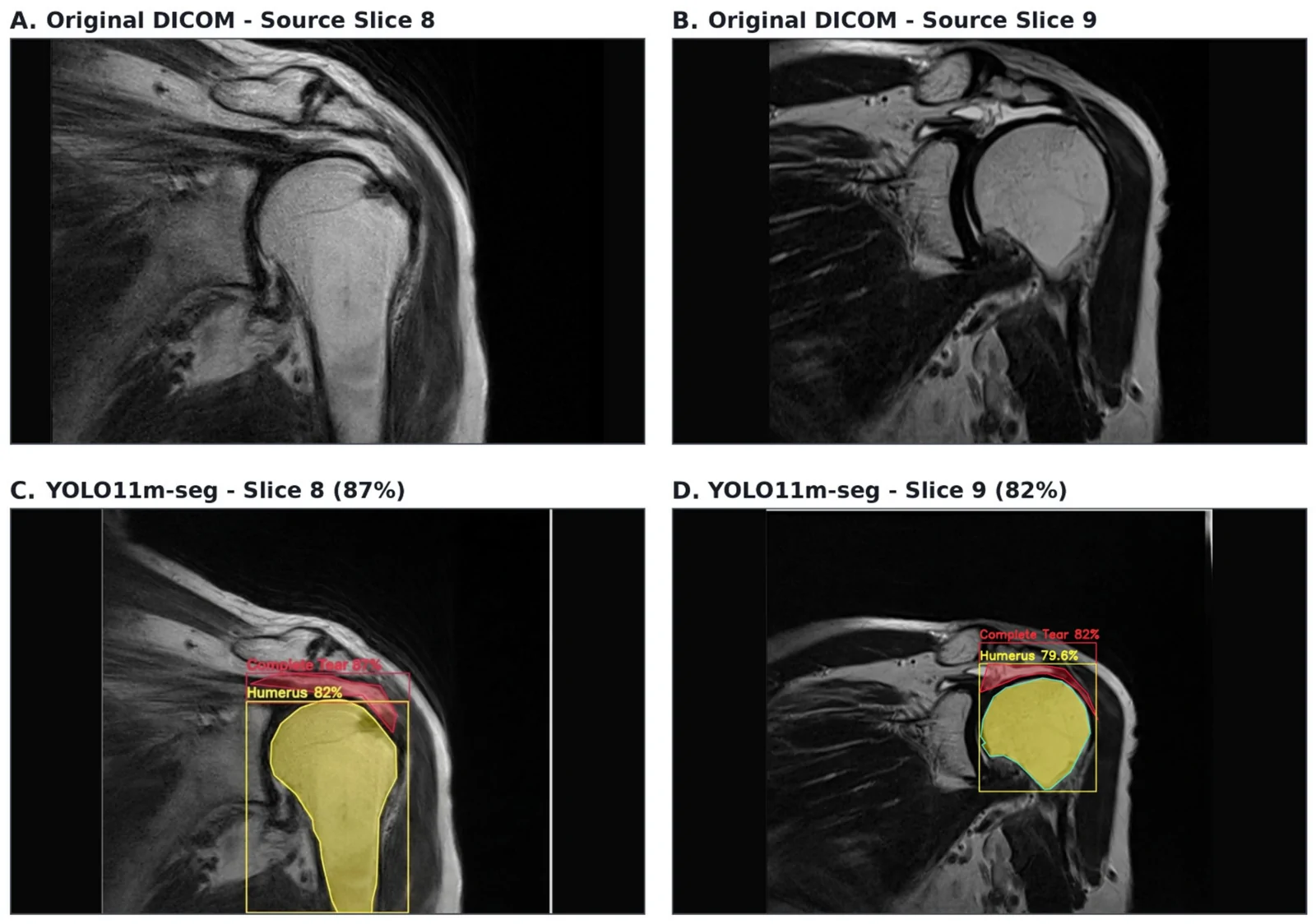
Representative author-reported source-slice and YOLO11m-seg inference images illustrating the intended two-distinct-adjacent-source-slice rule. (**A**,**B**) Source slices 8 and 9 from one deidentified shoulder examination, reported to have distinct pseudonymised source identifiers and anatomical positions. (**C**,**D**) Inference overlays with Complete Tear confidence scores of 0.87 and 0.82, respectively (threshold, 0.50). Repeated cine frames originating from the same anatomical source slice were not considered independent slices under the specified rule. Humerus was an auxiliary localisation class. This illustrative author-verified record does not independently establish correct execution of the aggregation rule across all 258 clinical cases.

For each reader-study case, the standardised cine derived from the complete coronal T2-weighted fat-suppressed sequence was supplied to the locked YOLO11m application. In the authors’ technical description, one representative decoded frame from the first playback was mapped to each of the 17–21 distinct original DICOM source slices for AI inference, rather than treating all 600 encoded video frames as independent MRI observations. Source-slice identity and anatomical order were described as being established from original DICOM identifiers and position metadata. The term “slice” in the prespecified patient-level aggregation rule therefore refers to a distinct original MRI source slice, not to an encoded video frame; repeated frames showing the same source image do not constitute independent anatomical evidence. The reported source-slice-to-frame mapping is illustrated by a representative case in Figure 3 and Supplementary Figure S1 (CASE-FT-001). The complete original cine-export and inference code was not available for independent verification of this implementation across the full cohort.

Predictions below the prespecified diagnostic confidence threshold of 0.50 were discarded. According to the authors’ technical documentation, inference used 640 × 640-pixel input images, a raw detection confidence floor of 0.001, and non-maximum suppression with an intersection-over-union threshold of 0.70; these settings could not be independently re-executed from the original application code. The diagnostic threshold of 0.50 and patient-level aggregation hierarchy were fixed during model development/validation and before evaluation of the arthroscopy-referenced cohort; neither was tuned using reader-study outcomes. The threshold is reported as a fixed study-specific operating point rather than as an independently established optimal threshold. When multiple diagnostic classes exceeded the threshold for the same source slice, the highest-confidence diagnostic class was retained for that slice. The auxiliary Humerus class was excluded from patient-level diagnostic aggregation.

Slice-level predictions were converted to one patient-level diagnosis using a prespecified hierarchical rule. A full-thickness tear required qualifying full-thickness predictions on at least two anatomically adjacent distinct source slices, as determined from the reported anatomical source-slice order. Two consecutive video frames derived from the same source slice did not satisfy this criterion. If the full-thickness criterion was not met, the case was classified as a partial-thickness tear when at least one qualifying partial-thickness prediction was present. An isolated full-thickness prediction was insufficient for full-thickness classification. Cases without a qualifying tear prediction, including those without any diagnostic prediction above the threshold, were classified as intact. The resulting priority was full-thickness tear, followed by partial-thickness tear, and then intact cuff.

The study authors supplied a representative deidentified source-slice/frame example and an aggregate technical audit summary as supplementary documentation of the intended rule and its reported implementation. These author-reported records do not include the complete case-level inference traces, executable source code, or trained model checkpoint and therefore do not enable independent reproduction of the original inference process.

### 2.6. Observer Assessment

Twelve orthopaedic physicians were assigned to four prespecified experience groups, with three readers each. Junior and senior residents had less than and at least 2.5 years of residency training, respectively. Junior specialists had less than five years of post-residency experience, whereas senior specialists had at least five years of post-residency experience and were actively involved in shoulder surgeries. The grouping variable was clinical seniority; detailed shoulder subspecialty exposure and individual MRI-reading volume were not collected as separate study variables.

The 258 cases were divided into three sets of 86 and evaluated sequentially during three sessions. Each reader independently assigned one of three patient-level diagnoses: intact cuff, partial-thickness tear, and full-thickness tear. Within each experience group, the prespecified panel diagnosis was the category supported by at least two of the three initial ratings. Three different initial diagnoses would have triggered reassessment under the planned protocol. According to the study authors’ verification of the archived first-pass reader summaries, all 258 cases in each panel received an initial majority diagnosis, with no three-way unresolved votes or reassessment rounds. Thus, first-pass and final panel classifications coincided. Individual first-pass reader accuracy and panel vote distributions are reported in Supplementary Tables S1 and S2, respectively. The complete case-level rating matrix was unavailable for independent verification in the present revision. The primary human comparator remained the prespecified three-reader majority panel rather than an individual physician.

Cases were presented in a randomised order using a standardised viewing protocol. Observers were blinded to clinical information, arthroscopic findings, operative records, AI predictions, and other observers’ assessments. Previous responses were not displayed, and no diagnostic feedback was provided.

A stratified 39-case subset of the arthroscopy-referenced reader cohort (13 cases per reference category) was designated for repeat assessment approximately one week after the primary evaluation. However, the paired reader-level first- and repeat-assessment records and coefficient-calculation outputs were not available for verification during this revision. Therefore, the previously reported intraobserver coefficients and repeat-agreement summaries were withdrawn rather than treated as verified results.

### 2.7. Statistical Analysis

The diagnostic performance of the 258 cases was assessed using accuracy, sensitivity, specificity, and F1 score. For the three-class task, the sensitivity, specificity, and F1 score were calculated using a one-versus-rest approach and reported as macro-averaged values. Accuracy confidence intervals were calculated using the Wilson method.

AI and each prespecified three-reader panel were compared on the same 258 cases using two-sided exact McNemar tests based on paired case-level correctness. Holm adjustment was applied separately to the four overall comparisons and the 12 class-specific comparisons. During revision, the paired tests and Holm adjustment were rechecked from the archived case-level results so that the multiplicity families matched these prespecified sets; the corrected adjusted *p* values are reported consistently in the Abstract, Results, tables, figures, Discussion, and Conclusions. Holm-adjusted *p* values were calculated from the unrounded exact *p* values.

Paired accuracy differences were defined as AI accuracy minus physician-panel accuracy in the same patients and expressed in percentage points. According to the authors’ retained paired-analysis results, unadjusted 95% confidence intervals for these differences were calculated using 20,000 patient-level paired nonparametric bootstrap resamples (percentile method; random seed 20261016). These intervals were not adjusted for multiple comparisons and were interpreted alongside, rather than in place of, Holm-adjusted exact McNemar tests. Discordant correctness counts (AI correct/panel incorrect and AI incorrect/panel correct), exact two-sided *p* values, and Holm-adjusted *p* values for each overall and class-specific paired comparison are provided in Supplementary Table S4.

First-pass within-panel agreement in the full 258-case cohort was described using the number of unanimous three-reader votes and two-of-three majority votes (Supplementary Table S2). The pairwise raw exact agreement in Table S2 represents three reader-pair comparisons per patient (774 comparisons per panel), without correction for chance. Chance-corrected interobserver coefficients from the prior analysis were not retained because they could not be reconciled with the authors’ verified first-pass vote distributions and the original case-level rating matrix and calculation code were unavailable for independent reanalysis. No intraobserver reliability coefficients were retained because the first–repeat rating records or sufficient calculation output could not be retrieved to substantiate them. Primary diagnostic analyses were conducted in Python 3.10. All hypothesis tests were two-sided, with adjusted *p* < 0.05 considered statistically significant.

## 3. Results

The reader-study cohort comprised 258 patients: 60 (23.3%) with an intact posterosuperior rotator cuff, 86 (33.3%) with a partial-thickness tear, and 112 (43.4%) with a full-thickness tear according to the arthroscopic reference standard. On the held-out technical test subset, the locked model achieved a mask mAP@50 of 0.890, precision of 0.886, recall of 0.901, and F1 score of 0.891. These technical segmentation metrics should not be interpreted as patient-level arthroscopy-referenced diagnostic accuracy.

According to the authors’ revised clinical-cohort summary, mean age was 55.90 ± 7.96 years (range, 35–78 years); 186 patients (72.1%) were female and 72 (27.9%) were male. The operated shoulder was right-sided in 186 cases (72.1%) and left-sided in 72 (27.9%). Detailed patient-level frequencies of surgical indications were unavailable in the retained analytic summary.

The reported technical test mask metrics were pooled across four segmentation classes (Normal Cuff, Partial Tear, Complete Tear, and the auxiliary Humerus class). The Humerus class was not used for three-category patient-level diagnosis. Separate class-specific technical segmentation metrics could not be reliably recovered from the retained technical test summary.

In the arthroscopy-referenced reader cohort, AI correctly classified 217 of 258 cases (accuracy, 0.841; 95% CI, 0.792–0.881), with macro-averaged sensitivity, specificity, and F1 scores of 0.843, 0.917, and 0.840, respectively. The senior-specialist panel achieved the highest numerical accuracy (235/258, 0.911), but the paired difference from AI was not significant after Holm adjustment (adjusted *p* = 0.060). The junior-specialist panel had an accuracy of 0.822 (adjusted *p* = 0.649), whereas AI showed higher accuracy than the senior-resident panel (0.729; adjusted *p* = 0.005) and junior-resident panel (0.671; adjusted *p* < 0.001). These are panel-level comparisons and should not be interpreted as estimates of a single physician’s performance.

In paired overall comparisons, the AI-minus-panel accuracy differences were −6.98 percentage points (unadjusted 95% paired bootstrap CI, −12.79 to −1.16) versus the senior-specialist panel, +1.94 (−4.65 to +8.53) versus the junior-specialist panel, +11.24 (+4.65 to +17.83) versus the senior-resident panel, and +17.05 (+10.08 to +24.03) versus the junior-resident panel. These confidence intervals were not adjusted for multiple comparisons; the corresponding exact McNemar discordant counts, full-precision *p* values, and Holm-adjusted *p* values for all overall and class-specific comparisons are reported in Supplementary Table S4. Overall three-category classification performance is summarised in Table 1.

For full-thickness tears, AI achieved a one-versus-rest accuracy of 0.888 (95% CI, 0.843–0.921), sensitivity 0.902, specificity 0.877, and F1 score 0.874. Its accuracy was lower than that of the senior-specialist panel (0.961; adjusted *p* = 0.034), with no statistically significant difference from the junior-specialist (0.911; adjusted *p* = 1.000), senior-resident (0.822; adjusted *p* = 0.218), or junior-resident panels (0.802; adjusted *p* = 0.074) after adjustment across the 12 class-specific comparisons (Table 2).

For partial-thickness tears, AI achieved a one-versus-rest accuracy of 0.849 (95% CI, 0.800–0.887), sensitivity 0.709, specificity 0.919, and F1 score 0.758. Its accuracy did not differ significantly from that of the senior-specialist (0.911; adjusted *p* = 0.259) or junior-specialist panels (0.833; adjusted *p* = 1.000), but exceeded that of the senior-resident (0.744; adjusted *p* = 0.034) and junior-resident panels (0.682; adjusted *p* < 0.001). Partial-thickness tears were the lowest-sensitivity category for AI (Table 3).

**Figure 4:**
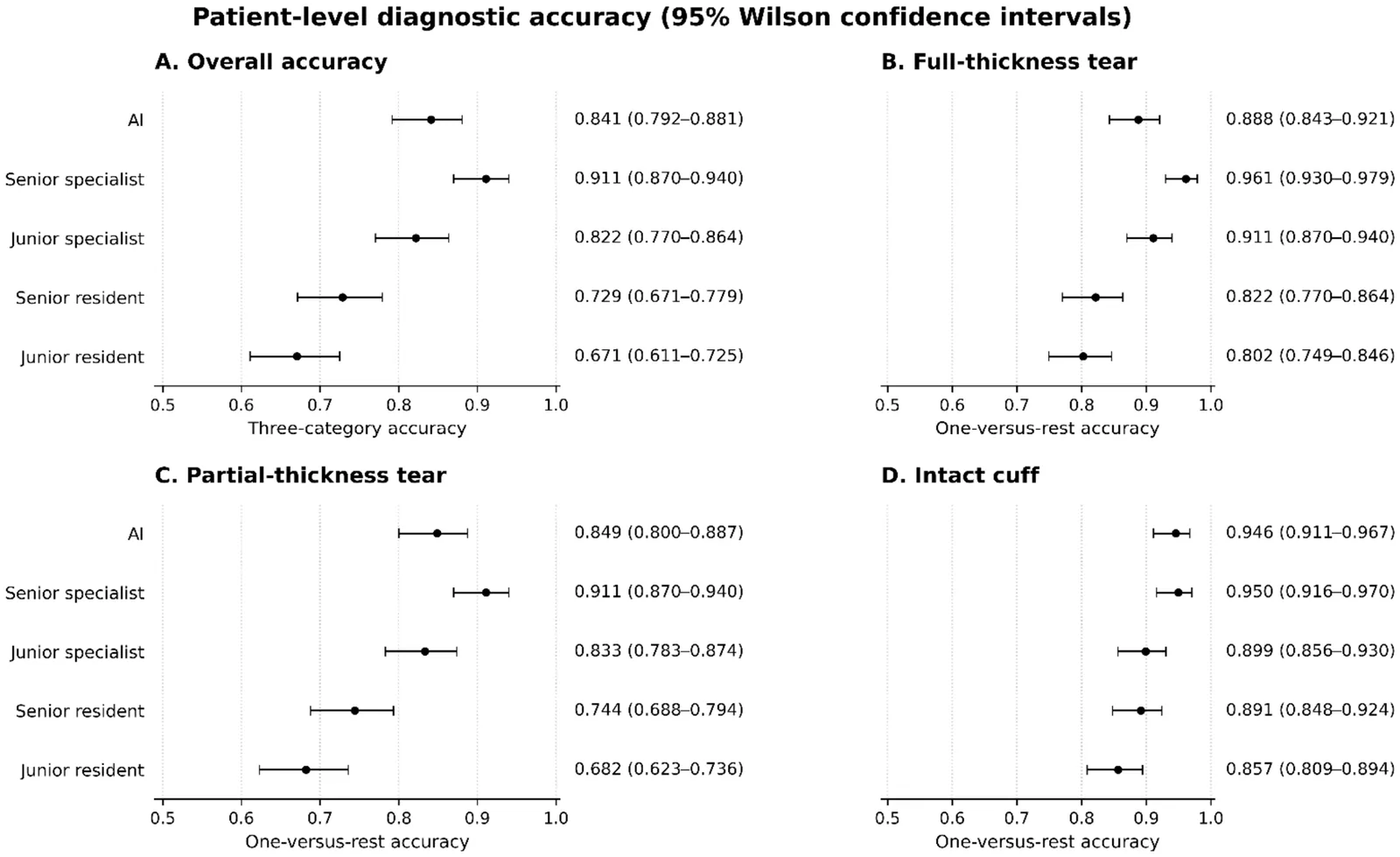
Overall three-category accuracy (**A**) and class-specific one-versus-rest accuracy for full-thickness tears (**B**), partial-thickness tears (**C**), and intact cuffs (**D**) for the AI model and the four prespecified three-reader majority panels (*n* = 258). The points represent accuracy and the bars show 95% Wilson confidence intervals. One-versus-rest accuracy is not equivalent to class-specific sensitivity. The values were calculated from the confusion matrices in Supplementary Table S3.

For intact posterosuperior rotator cuffs, AI achieved a one-versus-rest accuracy of 0.946 (95% CI, 0.911–0.967), sensitivity 0.917, specificity 0.955, and F1 score 0.887. Its accuracy did not differ significantly from those of the senior-specialist (0.950; adjusted *p* = 1.000), junior-specialist (0.899; adjusted *p* = 0.323), or senior-resident panels (0.891; adjusted *p* = 0.202), but exceeded that of the junior-resident panel (0.857; adjusted *p* = 0.007). Overall and class-specific accuracy estimates are shown in Figure 4 and Table 1, Table 2, Table 3 and Table 4.

The AI three-category confusion matrix is shown in Table 5. Among the 86 arthroscopically confirmed partial-thickness tears, AI correctly classified 61; the remaining 25 were classified as intact in eight cases and as full thickness in 17 cases. Among 112 full-thickness tears, 101 were correctly classified, 10 were classified as partial thickness, and one was classified as intact. Of 60 intact cuffs, 55 were correctly classified, four were classified as partial thickness, and one was classified as full thickness.

The four physician-panel three-category confusion matrices are reported in Supplementary Table S3. In the authors’ aggregate technical audit, all 119 cases assigned a full-thickness diagnosis by AI were reported to satisfy the required two-distinct-adjacent-source-slice rule, with no identified violations. The audit reported zero cases without a qualifying above-threshold diagnostic detection, three cases with isolated Complete Tear detections but no qualifying adjacent-slice pair, and zero cases with conflicting above-threshold diagnostic classes on the same source slice. All three isolated-positive cases were classified as partial-thickness tears by the AI hierarchy and had partial-thickness tears at arthroscopy. Among the 119 AI full-thickness predictions, the arthroscopic diagnoses were full-thickness in 101, partial-thickness in 17, and intact in one case. These are author-reported aggregate findings, not an independent rerun or complete case-level verification of the original inference software.

In the 258-case cohort, the first-pass panel votes were unanimous in 252, 255, 257, and 257 cases for the junior-resident, senior-resident, junior-specialist, and senior-specialist panels, respectively; the remaining six, three, one, and one cases were resolved by a two-of-three majority (Supplementary Table S2). No three-way unresolved first-pass votes or panel-level reassessment rounds were reported; first-pass and final panel diagnoses therefore coincided. Individual first-pass reader accuracies are presented in Supplementary Table S1. These counts describe unadjusted first-pass agreement rather than chance-corrected interobserver reliability. The previously reported first–repeat-agreement summaries and intraobserver coefficients were withdrawn because their underlying paired ratings and calculation records could not be supplied for verification.

## 4. Discussion

The main diagnostic finding was that the locked YOLO11m model achieved 0.841 three-category accuracy, with substantial variation according to panel experience and tear class. AI was numerically less accurate than the senior-specialist panel, but this paired difference did not meet the prespecified significance threshold after the Holm correction. The AI was more accurate than both resident panels, and the junior-specialist panel did not differ significantly from the AI. Partial-thickness tears remain the principal diagnostic limitation. These results describe the standalone performance against fixed three-reader majority panels, not the average individual-physician performance or AI-assisted clinical benefit.

For overall classification, AI accuracy was 0.841 despite strong segmentation performance on the held-out technical test subset, emphasising that mask-based technical scores and patient-level diagnoses are distinct outcomes. The numerical ordering of panel accuracies broadly resembles reports of AI-versus-reader performance in shoulder MRI [6,7,8], but direct comparison is limited by tendon definitions, image inputs, reference standards, and the use of panels rather than individual observers; heterogeneous study designs have also been highlighted in systematic reviews [10]. The senior-specialist panel had a higher point-estimate accuracy than AI, although the matched difference did not remain statistically significant after correction. Non-significance should not be interpreted as evidence of equivalence or non-inferiority.

Full-thickness tears showed the strongest tear-detection performance, with an AI sensitivity of 0.902 and an F1 score of 0.874. This pattern is concordant with that of Lin et al., whose model achieved the highest AUCs for full-thickness tears, and with Yao et al., who reported greater sensitivity for full-thickness than partial-thickness supraspinatus tears [8,18]. Conventional MRI studies have also shown high accuracy and limited observer variation for full-thickness defects [19]. The relatively conspicuous loss of tendon continuity and trans-tendinous fluid signal may provide more consistent features across adjacent slices, which is compatible with our prespecified consecutive-source-slice rule. Automated three-dimensional work has also demonstrated the feasibility of reproducible full-thickness tear localisation and measurement [11].

The principal diagnostic weakness of the model was the presence of partial-thickness tears. Although the class-specific accuracy was 0.849, the sensitivity was only 0.709, and the F1 score was 0.758. A similar difficulty has been reported in both AI-based and conventional MRI studies [13,18,20]. Incomplete fibre disruption, variable tear sidedness, and overlap with degenerative intratendinous signals likely reduce separability from both intact and full-thickness states [19,21]. Future work should specifically enrich arthroscopy-referenced partial-thickness cases, use more granular tear annotations, and evaluate complete multiplanar or multisequence inputs. Sequence-aware or three-dimensional models that integrate information across adjacent source slices may also improve sensitivity for subtle partial-thickness abnormalities.

For intact cuffs, AI achieved a sensitivity of 0.917 and a specificity of 0.955. The high one-versus-rest accuracy should be interpreted in light of the smaller intact class and the larger number of non-intact cases. No statistically significant accuracy differences were detected between AI and either the specialist panel or the senior-resident panel, whereas AI accuracy was higher than that of the junior-resident panel. The prespecified hierarchical rule may have supported this result by requiring a qualifying tear prediction before a case could leave the intact pathway. However, the intact category was defined by the absence of a tear in all three posterosuperior tendons and should not be interpreted as exclusion of all other shoulder pathology.

Reader agreement requires separate interpretation. First-pass vote concordance in the 258-case cohort is a descriptive measure of within-panel agreement, not a chance-corrected interobserver reliability coefficient. Results from the 39-case repeat-reading component are not interpreted because supporting first–repeat case-level records and calculation outputs were unavailable for verification; previously reported intraobserver κ estimates and repeat-agreement percentages were therefore removed.

As the physicians and AI were evaluated independently, the present study did not test the effect of AI assistance. Nevertheless, the results provide a rationale for prospective assisted reader studies, particularly among less experienced readers. Such studies should evaluate whether access to model output improves diagnostic accuracy, interpretation time, confidence, error patterns, and clinical decision-making, rather than assuming benefits from standalone performance alone.

The study had several limitations. Its retrospective single-centre design and restriction to arthroscopy-treated patients introduced selection, verification, and spectrum considerations; the observed class distribution may not represent an unselected shoulder MRI population. Detailed surgical indications, complete upstream screening counts, and the exact MRI-to-arthroscopy interval were not retained as analysable variables and could not be reconstructed reliably for this revision. All readers and the AI evaluated only a standardised coronal T2-weighted fat-suppressed cine rather than a complete clinical multiplanar MRI examination. Numerical acquisition parameters, including repetition/echo times, field of view, acquisition matrix, and slice thickness/inter-slice spacing, could not be recovered from the retained analytic materials. Arthroscopic reference categories were abstracted from operative reports rather than independently adjudicated operative videos. Operating surgeons had access to preoperative MRI during routine care, potentially introducing expectation or review bias. The development cohort used MRI-consensus labels without arthroscopic verification, and its curated slice selection may limit generalisability to unrestricted MRI series. The held-out development test was an internal mask-based technical assessment rather than an external clinical validation cohort. The retained technical test summary supported the prespecified pooled segmentation metrics but not a complete class-specific technical audit; additional class-specific technical test outputs were therefore not reconstructed post hoc. The representative source-slice mapping and authors’ aggregate technical audit did not permit independent verification of the two-distinct-adjacent-source-slice implementation across all 258 cases because the original executable inference code, complete patient-level traces, and model checkpoint were not available for independent review in this revision. Chance-corrected interobserver reliability could not be reported reliably because previously calculated values were inconsistent with the verified first-pass panel vote summaries, and the original rating matrix and calculation code were not available for reconciliation. The original paired first–repeat ratings and calculation records for the 39-case subset could not be retrieved for independent verification; accordingly, the intraobserver reliability coefficients and repeat-agreement estimates were removed from this revision. Finally, the study did not evaluate continuous patient-level scores, ROC calibration, decision-curve utility, or AI-assisted reading.

## 5. Conclusions

In this single-centre, arthroscopy-referenced three-category MRI study, YOLO11m-based AI showed higher paired accuracy than both resident panels, whereas neither specialist panel comparison reached the adjusted significance threshold. Partial-thickness tears remain the principal diagnostic limitation. These findings are specific to a standardised coronal-cine benchmark and do not establish equivalence to specialists or clinical benefits from AI assistance. External validation with complete multiplanar MRI and prospective assisted reader studies is required before clinical implementation of the model.

## Figures and Tables

**Table 1:** Overall three-category classification performance of AI and prespecified orthopaedic physician panels.

Panel/System	Accuracy	Sensitivity	Specificity	F1 Score	95% CI for Accuracy	Exact *p* vs. AI	Holm-Adjusted *p*
AI	0.841	0.843	0.917	0.840	0.792–0.881	—	—
Senior-Specialist Panel	0.911	0.898	0.954	0.903	0.870–0.940	0.030	0.060
Junior-Specialist Panel	0.822	0.809	0.910	0.809	0.770–0.864	0.649	0.649
Senior-Resident Panel	0.729	0.714	0.858	0.725	0.671–0.779	0.002	0.005
Junior-Resident Panel	0.671	0.660	0.831	0.664	0.611–0.725	<0.001	<0.001

CI, confidence interval. Accuracy 95% CIs were calculated using the Wilson method. Sensitivity, specificity, and the F1 score were macro-averaged across the three diagnostic classes. Physician-panel values represent the prespecified final two-of-three majority diagnosis among three independent readers. Exact two-sided McNemar tests were used to compare each panel with AI on the same 258 cases. Holm adjustment was applied across the four overall comparisons and was calculated using unrounded exact *p* values.

**Table 2:** Full-thickness tear classification performance of AI and prespecified orthopaedic physician panels.

Panel/System	Accuracy	Sensitivity	Specificity	F1 Score	95% CI for Accuracy	Exact *p* vs. AI	Holm-Adjusted *p*
AI	0.888	0.902	0.877	0.874	0.843–0.921	—	—
Senior-Specialist Panel	0.961	0.973	0.952	0.956	0.930–0.979	0.003	0.034
Junior-Specialist Panel	0.911	0.911	0.911	0.899	0.870–0.940	0.461	1.000
Senior-Resident Panel	0.822	0.795	0.842	0.795	0.770–0.864	0.036	0.218
Junior-Resident Panel	0.802	0.759	0.836	0.769	0.749–0.846	0.009	0.074

CI, confidence interval. Full-thickness tears were evaluated as a one-versus-rest binary outcome. Accuracy 95% CIs were calculated using the Wilson method. Physician-panel values represent the prespecified final two-of-three majority diagnosis among three independent readers. Exact two-sided McNemar tests were used to compare each panel with AI on the same 258 cases. Holm adjustment was applied across the 12 class-specific AI-versus-panel comparisons and was calculated using unrounded exact *p* values.

**Table 3:** The performance of AI and prespecified orthopaedic physician panels in the classification of partial-thickness tears.

Panel/System	Accuracy	Sensitivity	Specificity	F1 Score	95% CI for Accuracy	Exact *p* vs. AI	Holm-Adjusted *p*
AI	0.849	0.709	0.919	0.758	0.800–0.887	—	—
Senior-Specialist Panel	0.911	0.872	0.930	0.867	0.870–0.940	0.052	0.259
Junior-Specialist Panel	0.833	0.733	0.884	0.746	0.783–0.874	0.724	1.000
Senior-Resident Panel	0.744	0.698	0.767	0.645	0.688–0.794	0.004	0.034
Junior-Resident Panel	0.682	0.570	0.738	0.544	0.623–0.736	<0.001	<0.001

CI, confidence interval. Partial-thickness tears were evaluated as a one-versus-rest binary outcome. Accuracy 95% CIs were calculated using the Wilson method. Physician-panel values represent the prespecified final two-of-three majority diagnosis among three independent readers. Exact two-sided McNemar tests were used to compare each panel with AI on the same 258 cases. Holm adjustment was applied across the 12 class-specific AI-versus-panel comparisons and was calculated using unrounded exact *p* values.

**Table 4:** Intact-cuff classification performance of AI and prespecified orthopaedic physician panels.

Panel/System	Accuracy	Sensitivity	Specificity	F1 Score	95% CI for Accuracy	Exact *p* vs. AI	Holm-Adjusted *p*
AI	0.946	0.917	0.955	0.887	0.911–0.967	—	—
Senior-Specialist Panel	0.950	0.850	0.980	0.887	0.916–0.970	1.000	1.000
Junior-Specialist Panel	0.899	0.783	0.934	0.783	0.856–0.930	0.081	0.323
Senior-Resident Panel	0.891	0.650	0.965	0.736	0.848–0.924	0.029	0.202
Junior-Resident Panel	0.857	0.650	0.919	0.678	0.809–0.894	<0.001	0.007

CI, confidence interval. Intact cuffs were evaluated as a one-versus-rest binary outcome. Accuracy 95% CIs were calculated using the Wilson method. Physician-panel values represent the prespecified final two-of-three majority diagnosis among three independent readers. Exact two-sided McNemar tests were used to compare each panel with AI on the same 258 cases. Holm adjustment was applied across the 12 class-specific AI-versus-panel comparisons and was calculated using unrounded exact *p* values.

**Table 5:** Three-category confusion matrix for AI predictions against arthroscopic reference standard.

Arthroscopic Reference	Predicted Intact Cuff	Predicted Partial-Thickness Tear	Predicted Full-Thickness Tear
Intact Cuff	55	4	1
Partial-Thickness Tear	8	61	17
Full-Thickness Tear	1	10	101

The rows represent arthroscopic reference categories, and the columns represent AI patient-level predictions.

## Data Availability

Patient-level imaging and clinical records are not publicly available because of institutional and ethical restrictions. De-identified analytic data may be considered on reasonable request, subject to applicable institutional and/or ethics approval. The trained YOLO11m-seg checkpoint and original inference application source code are retained by the study’s AI-development team but are not publicly distributed because they constitute commercially sensitive intellectual property. The executable pipeline was not made available for independent evaluation during the present revision. The submitted representative source-slice evidence and author-reported aggregate technical audit do not include complete patient-level inference traces and do not permit independent reproduction of all 258 case-level classifications.

## Supplementary Materials

The following supporting information can be downloaded at https://www.mdpi.com/article/10.3390/diagnostics16213535/s1, Table S1, individual first-pass reader accuracy; Table S2, first-pass physician-panel vote distributions and descriptive agreement; Table S3, three-category confusion matrices for AI and all four physician panels; Table S4, paired AI–panel comparisons with McNemar discordances, exact and Holm-adjusted *p* values, accuracy differences, and confidence intervals; Supplementary Case Evidence CASE-FT-001, a representative deidentified source-slice/frame record; and an aggregate technical audit summary, author-reported results of the inference-rule assessment. A completed CLAIM 2024 reporting checklist is provided separately.
