# Supplementary material for: YOLO11m-Based Artificial Intelligence Versus Orthopaedic Physician Panels for Arthroscopy-Referenced Three-Category MRI Classification of the Posterosuperior Rotator Cuff: A Multi-Reader Diagnostic Accuracy Study
Source: Diagnostics (Basel). 2026 Oct 28;16(21):3535. doi: 10.3390/diagnostics16213535 (PMC13761931; doi:10.3390/diagnostics16213535)
Supplement: Supplementary file 1 [file diagnostics-16-03535-s001.zip › 02_Supplementary_Tables_S1-S4.pdf]

Supplementary Tables S1–S4

Manuscript diagnostics-4593904 | AI versus orthopaedic physician panels (n = 258)

Tables S1–S4 present the individual-reader accuracy, panel-vote distributions, confusion matrices, and paired AI–panel diagnostic accuracy comparisons for the arthroscopy-referenced cohort. The separate 39-case initial–repeat ratings are not included in these tables.

Table S1: First-pass individual-reader three-category diagnostic accuracy

Experience group	Reader ID	Correct (of 258)	Accuracy (%)
Junior resident	1	171	66.28
Junior resident	2	171	66.28
Junior resident	3	171	66.28
Senior resident	1	187	72.48
Senior resident	2	187	72.48
Senior resident	3	187	72.48
Junior specialist	1	211	81.78
Junior specialist	2	212	82.17
Junior specialist	3	212	82.17
Senior specialist	1	234	90.70
Senior specialist	2	235	91.09
Senior specialist	3	235	91.09

Table S2: First-pass panel votes and descriptive pairwise agreement

Experience group	3/3 votes	2/3 votes	Unresolved	Reassessment rounds	Panel correct	Pairwise exact agreement
Junior resident	252	6	0	0	173/258	762/774 (98.45%)
Senior resident	255	3	0	0	188/258	768/774 (99.22%)
Junior specialist	257	1	0	0	212/258	772/774 (99.74%)
Senior specialist	257	1	0	0	235/258	772/774 (99.74%)

Data provenance: The individual-reader and panel-vote counts below are unchanged from the authors' verified S1–S2 summaries. Exact pairwise agreement is calculated deterministically from the three-reader vote distribution; no patient-level ratings were imputed.

Accuracy is the number of exact matches to the arthroscopic reference category divided by 258. Each physician supplied one independent first-pass classification per patient. Individual-reader results are descriptive; primary physician comparisons use three-reader majority panels.

Panel decision: at least two of three matching initial diagnoses. "Unresolved" denotes three different initial diagnoses. No unresolved cases or reassessment rounds were reported in the authors' verified first-pass panel summaries.

Calculation: With U unanimous cases and D two-of-three majority cases, each unanimous case contributes three concordant reader pairs, and each 2/3 case contributes one concordant reader pair. Across 258 cases and three distinct reader pairs, exact pairwise agreement = (3U + D) / (3 × 258). The denominator is 774 reader pairs for each panel.

The pairwise agreement percentages are unweighted, unadjusted observed agreement across the full 258-patient cohort. They are NOT Gwet AC2, Cohen's kappa, or the interobserver agreement of the separate 39-patient repeat-reading subset. Individual first-pass rating records would be required to compute chance-corrected AC2 reliably.

Table S3: Three-category confusion matrices

AI model

Reference	Pred. intact	Pred. partial	Pred. full	Total
Intact cuff	**55**	4	1	60
Partial-thickness tear	8	**61**	17	86
Full-thickness tear	1	10	**101**	112
Total	64	75	119	258

Senior specialist panel

Reference	Pred. intact	Pred. partial	Pred. full	Total
Intact cuff	**51**	9	0	60
Partial-thickness tear	4	**75**	7	86
Full-thickness tear	0	3	**109**	112
Total	55	87	116	258

Junior specialist panel

Reference	Pred. intact	Pred. partial	Pred. full	Total
Intact cuff	**47**	13	0	60
Partial-thickness tear	10	**63**	13	86
Full-thickness tear	3	7	**102**	112
Total	60	83	115	258

Senior resident panel

Reference	Pred. intact	Pred. partial	Pred. full	Total
Intact cuff	**39**	20	1	60
Partial-thickness tear	4	**60**	22	86
Full-thickness tear	3	20	**89**	112
Total	46	100	112	258

Junior resident panel

Reference	Pred. intact	Pred. partial	Pred. full	Total
Intact cuff	**39**	20	1	60
Partial-thickness tear	14	**49**	23	86
Full-thickness tear	2	25	**85**	112
Total	55	94	109	258

Rows: arthroscopy reference. Columns: patient-level diagnosis. Bold diagonal cells denote correct predictions.

Panels are the first-pass two-of-three majority votes of three readers per experience group. The 258-patient reference distribution is 60 intact, 86 partial-thickness, and 112 full-thickness tears. Aggregated matrices cannot reconstruct paired AI–physician discordance counts; see Table S4.

Supplementary Table S4. Paired AI–physician diagnostic accuracy

A. Overall three-category accuracy (n = 258)

Endpoint	Physician panel	AI correct	Panel correct	b	c	AI–panel pp (95% CI)	Exact P	Holm P
Overall	Senior specialist	217/258	235/258	22	40	−6.98 [−12.79, −1.16]	0.0300158643491	0.0600317286981
Overall	Junior specialist	217/258	212/258	41	36	+1.94 [−4.65, +8.53]	0.648791162311	0.648791162311
Overall	Senior resident	217/258	188/258	55	26	+11.24 [+4.65, +17.83]	0.00168863807272	0.00506591421816
Overall	Junior resident	217/258	173/258	66	22	+17.05 [+10.08, +24.03]	2.87939074954e−06	1.15175629981e−05

B. Class-specific one-versus-rest accuracy (n = 258)

Endpoint	Physician panel	AI correct	Panel correct	b	c	AI–panel pp (95% CI)	Exact P	Holm P
Full-thickness	Senior specialist	229/258	248/258	10	29	−7.36 [−12.02, −2.71]	0.00337784791191	0.0337784791191
Full-thickness	Junior specialist	229/258	235/258	20	26	−2.33 [−7.36, +2.71]	0.461391182137	1
Full-thickness	Senior resident	229/258	212/258	38	21	+6.59 [+0.78, +12.40]	0.0363431799571	0.218059079742
Full-thickness	Junior resident	229/258	207/258	44	22	+8.53 [+2.33, +14.73]	0.00921048969455	0.0736839175564
Partial-thickness	Senior specialist	219/258	235/258	22	38	−6.20 [−12.02, −0.39]	0.0518938959289	0.259469479645
Partial-thickness	Junior specialist	219/258	215/258	38	34	+1.55 [−5.04, +8.14]	0.723948145747	1
Partial-thickness	Senior resident	219/258	192/258	54	27	+10.47 [+3.88, +17.05]	0.00359634771882	0.0337784791191
Partial-thickness	Junior resident	219/258	176/258	64	21	+16.67 [+10.08, +23.26]	3.27647035344e−06	3.93176442413e−05
Intact cuff	Senior specialist	244/258	245/258	13	14	−0.39 [−4.26, +3.49]	1	1
Intact cuff	Junior specialist	244/258	232/258	26	14	+4.65 [+0.00, +9.30]	0.080690467752	0.322761871008
Intact cuff	Senior resident	244/258	230/258	25	11	+5.43 [+0.78, +10.08]	0.0288167196559	0.201717037591
Intact cuff	Junior resident	244/258	221/258	33	10	+8.91 [+3.88, +13.95]	0.000606106631267	0.00666717294393

Definitions. b: AI correct/panel incorrect; c: AI incorrect/panel correct. Accuracy difference is AI minus panel in percentage points (pp). Exact McNemar P is a two-sided conditional binomial test. Holm adjustment was applied separately to the 4 overall and 12 class-specific comparisons, using full-precision P values.

Unadjusted 95% paired-difference CIs: 20,000 patient-level bootstrap resamples (percentile method; random seed 20261016). Class-specific accuracy is one-versus-rest accuracy, not class sensitivity. The 258-case historical paired analysis was verified by the authors; patient-level matched outputs were unavailable for independent reconstruction. Discordant b/c counts cannot be reconstructed from Table S3 margins alone.
