# Supplementary material for: YOLO11m-Based Artificial Intelligence Versus Orthopaedic Physician Panels for Arthroscopy-Referenced Three-Category MRI Classification of the Posterosuperior Rotator Cuff: A Multi-Reader Diagnostic Accuracy Study
Source: Diagnostics (Basel). 2026 Oct 28;16(21):3535. doi: 10.3390/diagnostics16213535 (PMC13761931; doi:10.3390/diagnostics16213535)
Supplement: Supplementary file 1 [file diagnostics-16-03535-s001.zip › 03_CASE-FT-001_SourceSlice_Evidence.pdf]

YOLO11m Positive Full-Thickness Case Evidence

1. Evidence package and data lineage

This case-level record presents actual inference results and demonstrates the DICOM-to-cine frame mapping and source-slice aggregation process in a representative patient. The original MRI sequence comprises 20 distinct DICOM source slices, represented by 600 encoded video frames across two consecutive 10-second playback cycles at 30 frames per second.

Property	Record
Case identifier	CASE-FT-001
Target architecture	YOLO11m-seg
Distinct source slices	20
Video playback	2 cycles; 10 s each; 30 fps
Encoded video frames	600
Inference unit	One unique source slice
Threshold	0.50
Case-level decision	Full-thickness tear, based on source slices 8 and 9

2. DICOM-to-cine relationship

Each original source-slice index is associated with a distinct pseudonymous source identifier. The cine conversion assigns 15 video frames per slice per playback cycle. As both cycles repeat the same source slices, 30 presentation frames are mapped to one unique source image; those repetitions cannot independently establish anatomical adjacency.

Video frame interval	Cycle	Source slice	Source identifier prefix
105–119	1	8	70bff6a4f472c58c
120–134	1	9	635047a397c11b60
405–419	2	8	70bff6a4f472c58c
420–434	2	9	635047a397c11b60

The table above presents representative frame intervals from both playback cycles, demonstrating how repeated encoded video frames map to distinct original DICOM source slices. Only selected mapping records are presented in this report.

3. Distinct adjacent source-slice proof

The diagnostic rule operates after encoded frame indices have been converted to unique source-slice identities. A pair qualifies only if both source indices are different, their anatomical order is consecutive and both pass the confidence threshold for the full-thickness class.

Criterion	Slice 8	Slice 9
Source-slice index	8	9
Source ID	70bff6a4f472c58c	635047a397c11b60
Position along normal	49.4 mm	53.6 mm
Complete Tear score	0.87	0.82
Qualifying score	Yes	Yes
Representative frame (first cycle)	112	127

The source identifiers are distinct, the consecutive index difference is one and both confidence values are at least 0.50. Slice 4 (0.71) and Slice 13 (0.68) are isolated full-positive candidates that do not, by themselves, satisfy the two-slice criterion.

4. Whole-case source-slice prediction trace

Slice	Position (mm)	Diagnostic winner	Score
1	20.0	Normal Cuff	0.77
2	24.2	Normal Cuff	0.89
3	28.4	Normal Cuff	0.73
4	32.6	Complete Tear	0.71
5	36.8	Partial Tear	0.64
6	41.0	Normal Cuff	0.55
7	45.2	none	—
8	49.4	Complete Tear	0.87
9	53.6	Complete Tear	0.82
10	57.8	none	—
11	62.0	Normal Cuff	0.59
12	66.2	Normal Cuff	0.57
13	70.4	Complete Tear	0.68
14	74.6	none	—
15	78.8	Partial Tear	0.55
16	83.0	Normal Cuff	0.83
17	87.2	none	—
18	91.4	Normal Cuff	0.81
19	95.6	Normal Cuff	0.75
20	99.8	Normal Cuff	0.72

The winning class excludes the auxiliary Humerus category. Each row shows the highest-confidence qualifying diagnostic category for one original source slice, where present. No separate class-level detection table is included.

5. Patient-level aggregation

Rule component	Trace result
All full-positive source slices	4, 8, 9, 13
Qualifying adjacent full-positive pair	[8, 9]
Isolated full-positive source slices	[4, 13]
Partial-positive source slices	[5, 15]
Decision hierarchy	Full-thickness > partial-thickness > intact
Final case classification	full_thickness

6. Supplementary Figure S1 — original-source adjacency diagram

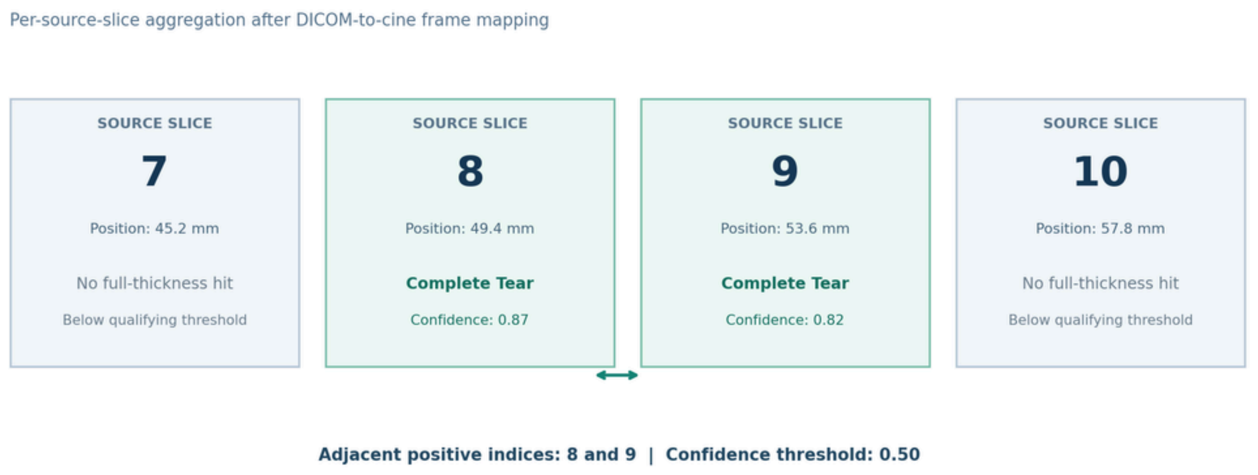

Supplementary Figure S1. Anatomically indexed source-slice trace for consecutive full-thickness scores. Source slices 8 and 9 have distinct source identifiers and are mapped to separate sets of encoded frames across both playback cycles.
