# Supplementary material for: YOLO11m-Based Artificial Intelligence Versus Orthopaedic Physician Panels for Arthroscopy-Referenced Three-Category MRI Classification of the Posterosuperior Rotator Cuff: A Multi-Reader Diagnostic Accuracy Study
Source: Diagnostics (Basel). 2026 Oct 28;16(21):3535. doi: 10.3390/diagnostics16213535 (PMC13761931; doi:10.3390/diagnostics16213535)
Supplement: Supplementary file 1 [file diagnostics-16-03535-s001.zip › 04_Aggregate_Technical_Audit.pdf]

AGGREGATE TECHNICAL AUDIT SUMMARY

Verification of the two-distinct-adjacent-source-slice rule for patient-level YOLO11m-seg classification

Study: Arthroscopy-referenced posterosuperior rotator cuff MRI cohort   N = 258   Manuscript: diagnostics-4593904

1. Scope and source of evidence

This report summarizes the study authors' aggregate technical verification findings from source-slice-level records retained during the original inference of the 258 clinical cases. The prespecified full-thickness decision required Complete Tear predictions with confidence ≥ 0.50 on two distinct, anatomically adjacent original DICOM source slices. Repeated encoded cine frames originating from the same source slice did not constitute independent anatomical evidence.

2. Full-cohort technical audit results

Verification parameter	Author-reported result
Arthroscopy-referenced cases evaluated	**258**
Cases assigned full-thickness tear by AI	**119**
AI full-thickness cases fulfilling the two-source-slice rule	**119 / 119**
AI full-thickness cases violating the two-source-slice rule	**0**
Patients with no qualifying diagnostic detection (confidence ≥ 0.50)	**0**
Patients with isolated full-thickness finding(s) but no qualifying adjacent pair	**3**
Final AI classification of these 3 isolated-positive cases	**Partial tear: 3 / 3**
Arthroscopic reference diagnosis of these 3 cases	**Partial tear: 3 / 3**
Patients with conflicting diagnostic-class predictions on the same source slice	**0**

Definition note: The three isolated-positive cases had one or more qualifying full-thickness source-slice detections, but no qualifying adjacent full-thickness pair. All three were classified as partial-thickness tears because qualifying partial-thickness predictions were present. The reported zero-conflict count refers specifically to competing diagnostic-class predictions on the same source slice; different categories on separate slices are not counted as within-slice conflicts.

3. Arthroscopic reference diagnoses among AI full-thickness classifications

Arthroscopic reference category	Cases
Intact cuff	**1**
Partial-thickness tear	**17**
Full-thickness tear	**101**
Total AI-classified full-thickness cases	**119**

The 119 cases above are AI-predicted full-thickness cases, not 119 surgically confirmed full-thickness tears. The reference-diagnosis distribution is taken from the archived 258-case patient-level confusion matrix.

4. Interpretation and disclosure boundary

According to the authors' audit of the original inference-time records, all 119 AI full-thickness classifications met the required adjacent-source-slice criterion, with no identified rule violations. The separate representative-patient YOLO11m Positive Full-Thickness Case Evidence report provides an illustrative original-case frame-to-source-slice mapping, including distinct source identifiers, anatomical positions, and confidence scores.

This document reports aggregate author-verified findings; it does not include the 258 individual patient inference traces, trained model weights, or proprietary application source code. Accordingly, the summary and the representative case evidence do not constitute independent reproduction of the original software execution or external verification of every patient record.
