# Supplementary material for: YOLO11m-Based Artificial Intelligence Versus Orthopaedic Physician Panels for Arthroscopy-Referenced Three-Category MRI Classification of the Posterosuperior Rotator Cuff: A Multi-Reader Diagnostic Accuracy Study
Source: Diagnostics (Basel). 2026 Oct 28;16(21):3535. doi: 10.3390/diagnostics16213535 (PMC13761931; doi:10.3390/diagnostics16213535)
Supplement: Supplementary file 1 [file diagnostics-16-03535-s001.zip › 05_CLAIM2024_Checklist.pdf]

CLAIM 2024 — Completed Reporting Checklist

Manuscript diagnostics-4593904 | Final publication proof | Locations refer to the manuscript sections, figures, tables, and named supplementary documents.

Status: YES = substantially reported at the specified location (qualified caveats may apply); NO = missing or insufficiently documented; NA = not applicable. This is a reporting checklist, not independent verification of patient-level data, AI inference execution, source code, or reader-level coefficients.

#	Concise CLAIM 2024 item	Status	Manuscript location	Reporting status / qualification
1	Identify AI methodology and technique	YES	Title; §2.4	YOLO11m-seg specified in Methods.
2	Structured abstract with design, cohorts, analysis and outcomes	YES	Abstract	Study and key accuracy results reported in structured abstract.
3	Clinical/scientific context and intended AI role	YES	§1 Introduction	Intended standalone comparator role described, not assisted-reading efficacy.
4	Objectives, hypothesis and research question	YES	§1 Introduction	Three diagnostic categories and panel comparators described.
5	Prospective versus retrospective design	YES	§2.1	Single-centre retrospective diagnostic study.
6	Study goal and target clinical prediction	YES	§§2.1, 2.5	Patient-level three-category tear diagnosis versus arthroscopy.
7	Data sources and their representativeness	YES	§§2.1, 2.4; Data Availability Statement	Development cohort MRI-consensus (300); clinical cohort arthroscopy (258); nonrepresentative surgical selection.
8	Inclusion/exclusion criteria and recruitment	YES	§2.1; Figure 1	Eligibility detailed; initial upstream screening denominator unavailable.
9	Image/data preprocessing and software settings	YES	§§2.3–2.5	Input, cine settings and inference configuration described; executable provenance not independently verified.
10	Selection or exclusion of image/data subsets	YES	§2.4	Peripheral and category-nondiagnostic images excluded from model development.
11	Methods for de-identification	NO	§2.3; Data Availability Statement	Anonymization stated; specific DICOM tags/procedure not fully reported.
12	Handling of missing data	NO	§2.1; §3 Results	All final 258 cases have complete analysis requirements; upstream missingness and exclusions incompletely reported.
13	Full imaging acquisition protocol	NO	§2.3	1.5 T and sequence reported; numeric TR/TE, gap, FOV, matrix, thickness unavailable.
14	Definition and acquisition of the reference standard	YES	§§2.2, 2.4	Arthroscopy for reader cohort; MRI-consensus for model development, not equated.
15	Justification, bias and limits of reference standard	YES	§2.2; §4 Discussion	Operative documentation standard; potential surgeon MRI-expectation bias acknowledged.
16	Reference-label sources and annotator expertise	YES	§§2.2, 2.4	Joint MRI development annotations; reference arthroscopy based on operative records.
17	Test-set annotation process and reproducible labeling	NO	§2.4; Figure 2	Original representative CVAT masks shown; full reusable labeled mask set not supplied.

#	Concise CLAIM 2024 item	Status	Manuscript location	Reporting status / qualification
18	Inter/intra-rater variability for reference labeling	NO	§§2.4, 2.6; §4 Discussion	Independent reliability of the development MRI-consensus reference labels was not assessed. The 39-case reader repeat-assessment component is described, but its coefficients are not reported in Round 3 because original paired calculation records were unavailable.
19	Training, optimization and testing allocation	YES	§2.4; Figure 1	210/45/45 patient-level allocation; image counts reported.
20	Disjointness of partitions and cohorts	YES	§§2.1, 2.4, 2.6; Figure 1	Development and clinical cohorts were nonoverlapping. The prespecified 39-case repeat-assessment subset was nested within the 258-case surgical cohort; no repeatability coefficients are reported.
21	Test set size and sample-size justification	YES	§§2.1, 2.4	Actual test set sizes described; no prospective sample-size calculation.
22	Detailed model architecture, inputs and outputs	NO	§§2.4–2.5	Architecture named, input and classes described; proprietary implementation details not sufficient for full replication.
23	Names and versions of training and inference software	YES	§§2.3–2.5; §2.7	Python 3.10, PyTorch 2.11.0, Ultralytics 8.3.0; original executable inference environment not available to reviewers.
24	Model initialization / transfer learning	YES	§2.4	COCO-pretrained segmentation weights reported.
25	Training procedures, losses and hyperparameters	NO	§2.4	Epochs, augmentation, AdamW, learning rate, batch size described; all losses/search history not documented.
26	Final model selection and checkpoint locking	YES	§2.4	Highest validation mask mAP@50; checkpoint frozen before clinical cohort.
27	Ensembling details, if applicable	NA	§2.4 (not applicable)	One model; no multi-model ensemble.
28	Performance metrics and relevant benchmarks	YES	§2.7; §3 Results; Tables 1–5	Overall and class-specific diagnostic performance; pooled mask technical metrics.
29	Statistical significance and uncertainty methods	YES	§2.7; §3 Results; Supplementary Table S4	Wilson confidence intervals, exact paired McNemar tests, Holm multiplicity adjustment, and paired bootstrap confidence intervals are described. Intraobserver coefficients were removed in Round 3 because their calculation records were not available for verification.
30	Robustness and sensitivity analyses	NO	§§2.5–2.7; §3 Results; Supplementary Table S2	No threshold or acquisition sensitivity study; first-pass majority equals final vote for all panels.
31	Explainability technique, if applied	NA	§2.5 (not applicable)	No separate XAI model evaluated; segmentation output is not an explainability validation.
32	Internal test procedure and model-performance comparison	NO	§2.4; §3 Results; §4 Discussion	45-case held-out technical test has pooled four-class mask metrics; per-class metrics unavailable.
33	External-site testing or acknowledged absence	YES	§4 Discussion	No independent institution testing; limitation is explicitly stated.
34	Clinical trial registration, where relevant	NA	§2.1 (not applicable)	Retrospective observational diagnostic study, not an interventional clinical trial.
35	Included and excluded counts, selection flow	NO	§2.1; Figure 1; §3 Results	258 final cases described; full screened and upstream excluded numbers not recoverable.

#	Concise CLAIM 2024 item	Status	Manuscript location	Reporting status / qualification
36	Demographic and clinical characteristics by dataset	YES	§3 Results	Age, sex, side summarized for clinical cohort; detailed indication and stratified demographics missing.
37	Model metrics across data partitions/subgroups	NO	§2.4; §3 Results	Only pooled technical metrics available; no complete technical class-by-class or subgroup metrics.
38	Diagnostic performance with uncertainty estimates	YES	§3 Results; Tables 1–5; Supplementary Table S4	Accuracy, class sensitivity/specificity/F1 and Wilson CIs; paired accuracy-difference CIs in S4.
39	Error analysis and confusion matrices	YES	§3 Results; Table 5; Supplementary Table S3	AI and four panel matrices in S3, AI matrix in Table 5; partial-tear error directions given.
40	Limitations, bias, and generalizability	YES	§4 Discussion	Retrospective, single-centre design; surgical selection; lack of external validation; incomplete executable source-slice provenance; and unavailable first–repeat reader calculation records are acknowledged.
41	Clinical role, implementation and future research	YES	§4 Discussion; §5 Conclusions	Standalone study limitation and prospective assisted-reading/external testing recommended.
42	Protocol or additional technical documentation access	NO	§2.5; Data Availability Statement; CASE-FT-001 and Aggregate Technical Audit	The CASE-FT-001 source-slice example and author-reported Aggregate Technical Audit Summary are provided as supplementary evidence. They do not furnish a fully independently executable pipeline or all case-level inference traces.
43	Availability statement for software, model and data	YES	Data Availability Statement	Software, data and proprietary restrictions disclosed; model and code not publicly shared.
44	Funding/support and funder roles	YES	Funding; Author Contributions	No external funding; author contributions stated; no funder role reported.

Item descriptions are concise paraphrases of the CLAIM 2024 reporting guideline and preserve the authors' 44-item reporting crosswalk. For the official wording, see the RSNA CLAIM 2024 checklist.
